# Seeing without touching: weak-disturbance imaging and characterization of ultra-confined optical near fields

**DOI:** 10.1038/s41377-025-02110-7

**Published:** 2026-01-03

**Authors:** Bowen Wang, Qian Chen, Chao Zuo

**Affiliations:** 1 Smart Computational Imaging Laboratory (SCILab), School of Electronic and Optical Engineering, Nanjing University of Science and Technology, Nanjing, Jiangsu Province 210094 China; 2 Jiangsu Key Laboratory of Visual Sensing & Intelligent Perception, Nanjing, Jiangsu Province 210094 China; 3 State Key Laboratory of Extreme Environment Optoelectronic Dynamic Measurement Technology and Instrument, Taiyuan, Shanxi Province 030051 China

**Keywords:** Nanophotonics and plasmonics, Sub-wavelength optics, Imaging and sensing

## Abstract

A recent study employing high-spatial-resolution photoemission electron microscopy (PEEM) achieved, for the first time, weak-disturbance imaging of the ultra-confined nanoslit mode in a coupled nanowire pair (CNP), revealing its quasi-three-dimensional field distribution.

As advances in photonics continue to push the boundaries of physical limits, a fundamental question is being revisited: *What is the ultimate limit of how tightly light can be confined?* Beyond this, *can we truly “witness” light at such an ultimate scale without disturbing it*? Achieving sub-wavelength or even atomic-scale confinement promises profound insights into nanoscale light–matter interactions^1^, with far-reaching implications for nonlinear optics^2–4^, super-resolution microscopy^5,6^, and next-generation photonic devices^7^. Recent progress in metallic and dielectric nanostructures has enabled optical field confinement down to sub-10-nm^8–11^ and even sub-1-nm^12–15^ scales, with applications ranging from nanowaveguides to nanolasers. However, in metallic plasmonic modes, when the optical field is confined to extremely small scales, the problems of momentum mismatch and material damage become pronounced^16,17^. In contrast, dielectric nanostructures^18^ exploit the coherent oscillation of polarized bound electrons to achieve extreme optical field confinement at sub-nm scales under low-loss conditions. In particular, sub-nm optical field confinement in a coupled nanowire pair (CNP) with a ~1-nm-wide slit has been demonstrated^13–15^. However, such fields are restricted to the near-field region of ultra-small feature sizes, where even minimal detector interaction can disturb their fragile near-field distribution. This challenge makes the pursuit of “observation without disturbance” both paradoxical and essential.

Conventional scanning near-field optical microscopy can characterize optical near fields at ~10-nm resolution^19–21^, but probing even more tightly confined fields requires reducing the probe–sample distance to the point where significant disturbance becomes unavoidable. Photoemission electron microscopy (PEEM), on the other hand, leverages the photoelectric effect^22^ to detect photoelectrons emitted from the illuminated surface (“photons in, electrons out”), offering high spatial resolution and sensitivity with minimal perturbation to the original field. These features make PEEM an ideal platform for imaging and characterizing ultra-confined near fields that are otherwise inaccessible to conventional near-field probes^23–25^.

In a recent study^26^ published in *Light: Science & Applications*, Prof. Limin Tong’s team at Zhejiang University, in collaboration with Guowei Lyu and Yaolong Li from Peking University reports, for the first time, weak-disturbance imaging of a sub-nm-confined nanoslit mode in a CNP using high-spatial-resolution PEEM. PEEM detects photoelectrons emitted via the photoelectric effect without the need for a physical probe, offering high sensitivity and minimal perturbation to the optical field. The experimental setup (Fig. 1a) employed a femtosecond laser beam, passed through a half-wave plate (HWP) for polarization control, and focused onto the CNP positioned on an indium tin oxide (ITO)-coated substrate. The inset in Fig. 1a shows the simulated electric field distribution of the TE₀-like nanoslit mode in the CNP cross-section. The CNP samples were fabricated by assembling single-crystal ZnO nanowires, grown via a bottom-up method to achieve atomically smooth surfaces, into structures that naturally formed uniform ~1 nm-wide slits between opposing facets. Morphological characterization (Fig. 1b), including scanning electron microscopy (SEM) and high-resolution transmission electron microscopy (HR-TEM), confirmed the precise nanoslit geometry. When vertically polarized femtosecond laser pulses were applied, the TE₀-like nanoslit mode, with its electric field concentrated in the slit, was successfully excited. PEEM imaging revealed a distinct standing-wave pattern localized at the mid-gap of the CNP, originating from coherent interference between the nanoslit mode and incident light guided along the nanowires. Simulations predicted field confinement of ~0.4 nm along the y-axis and ~4 nm along the z-axis (full width at half maximum, FWHM). While the PEEM-measured profile along y-axis was broadened to ~40 nm due to the limited instrumental resolution, the measured mode shape and effective wavelength closely matched theoretical predictions.

**Fig. 1:**
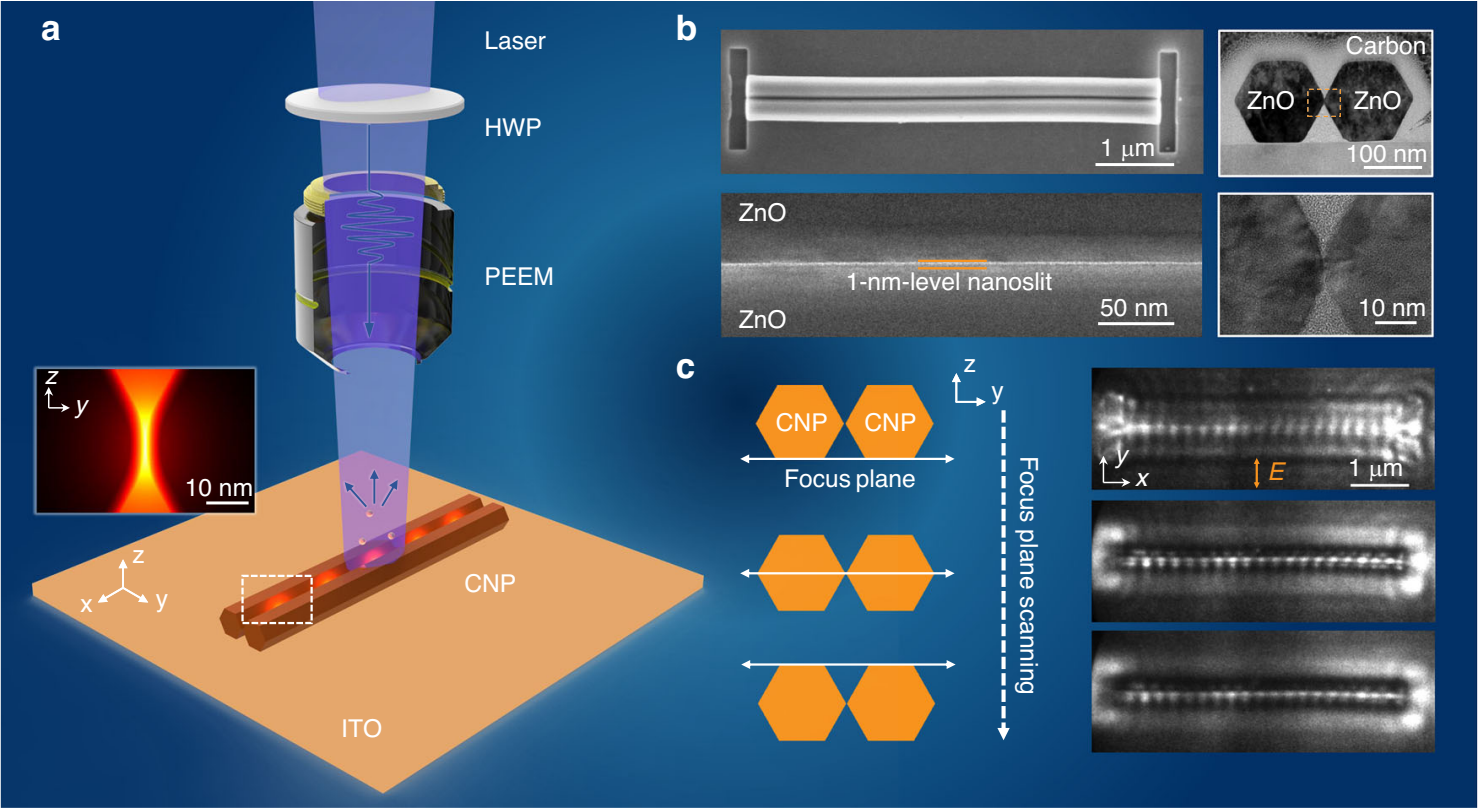
**Weak-disturbance imaging and quasi-3D characterization of ultra-confined nanoslit modes using PEEM**. **a** Schematic of PEEM experimental setup. A femtosecond laser beam passes through a half-wave plate (HWP) for polarization control and is focused onto the CNP placed on an indium tin oxide (ITO)-coated substrate; inset: simulated electric field distribution of the TE₀-like nanoslit mode in the CNP cross-section. **b** Morphological characterization of a ZnO CNP: top left, scanning electron microscopy (SEM) image; top right, cross-sectional high-resolution transmission electron microscopy (HR-TEM) image showing the hexagonal cross-section and the ~1 nm slit; bottom left, SEM image of the nanoslit region; bottom right, high-magnification HR-TEM image of the slit. **c** Quasi-three-dimensional imaging of the nanoslit mode by varying the focal plane along the z-axis: schematic of focal plane positions (left) and corresponding PEEM images (right), revealing maximum contrast at the CNP mid-gap for vertical polarization

To examine the nanoslit mode’s spatial distribution along the z-axis, the research team performed quasi-three-dimensional (3D) imaging by adjusting the focal plane of the PEEM system (Fig. 1c). When focused at the ITO substrate (the bottom of the CNP), the PEEM images appeared blurred for both vertical and horizontal polarizations. As the focal plane was raised to the CNP’s mid-plane, the contrast of the vertically polarized nanoslit mode reached its maximum, pinpointing the localized hotspot at the central slit. These observations provide direct experimental evidence of the nanoslit mode’s quasi-3D spatial distribution and highlight PEEM’s potential for reconstructing volumetric field distributions in nanostructures. Moreover, PEEM characterization proved highly sensitive to subtle structural variations in the CNP, such as slightly increased slit widths or non-uniform nanowire alignment, that can significantly alter photoemission intensity but are difficult to detect with conventional techniques like SEM or TEM.

Looking forward, although the current PEEM resolution (~40 nm) still limits direct visualization of sub-nm features and precludes direct retrieval of phase and vectorial information, its unique non-perturbative detection capability and high surface sensitivity make it a powerful tool for probing extreme optical fields. Future progress could come from several promising directions: (1) Integrating multi-angle illumination^27^ or focal-plane scanning^28^ to further enhance axial (z-axis) resolution and enable faithful 3D reconstruction of light-field distributions within bulk samples^29^; (2) Leveraging femtosecond time-resolved PEEM (TR-PEEM)^30,31^ to dynamically capture the electronic response during the formation, evolution, and annihilation of extreme optical fields, thereby revealing transient light-matter interaction mechanisms at the nanoscale; (3) Combining PEEM with computational imaging techniques, such as physics-based optical field inversion^32–34^ and deep learning-driven field reconstruction^35,36^, to further surpass intrinsic resolution and information-dimensionality limits, enabling recovery of nonlinear responses, excitation lifetimes, and other key physical quantities. Together, these advances could transform PEEM from a tool for “field intensity mapping” into a platform for “computational multidimensional sensing,” deepening our understanding of nanoscale light-matter interactions and providing essential characterization capabilities for the development of next-generation high-performance photonic chips, nanophotonic sensors, and optical information devices.

## References

[1] Betzig, E. & Trautman, J. K. Near-field optics: microscopy, spectroscopy, and surface modification beyond the diffraction limit. *Science* **257**, 189–195 (1992). 17794749 10.1126/science.257.5067.189

[2] Meng, Y. J. et al. Bright single-nanocrystal upconversion at sub 0.5 W cm^−2^ irradiance via coupling to single nanocavity mode. *Nat. Photonics* **17**, 73–81 (2023).

[3] Choi, H., Heuck, M. & Englund, D. Self-similar nanocavity design with ultrasmall mode volume for single-photon nonlinearities. *Phys. Rev. Lett.* **118**, 223605 (2017). 28621978 10.1103/PhysRevLett.118.223605

[4] Benz, F. et al. Single-molecule optomechanics in “picocavities”. *Science* **354**, 726–729 (2016). 27846600 10.1126/science.aah5243

[5] Siday, T. et al. All-optical subcycle microscopy on atomic length scales. *Nature* **629**, 329–334 (2024). 38720038 10.1038/s41586-024-07355-7

[6] Yang, B. et al. Sub-nanometre resolution in single-molecule photoluminescence imaging. *Nat. Photonics* **14**, 693–699 (2020).

[7] Atabaki, A. H. et al. Integrating photonics with silicon nanoelectronics for the next generation of systems on a chip. *Nature* **556**, 349–354 (2018). 29670262 10.1038/s41586-018-0028-z

[8] Wang, P. et al. Molecular plasmonics with metamaterials. *Chem. Rev.* **122**, 15031–15081 (2022). 36194441 10.1021/acs.chemrev.2c00333 PMC9562285

[9] Albrechtsen, M. et al. Nanometer-scale photon confinement in topology-optimized dielectric cavities. *Nat. Commun.* **13**, 6281 (2022). 36271087 10.1038/s41467-022-33874-w PMC9587274

[10] Babar, A. N. et al. Self-assembled photonic cavities with atomic-scale confinement. *Nature* **624**, 57–63 (2023). 38057568 10.1038/s41586-023-06736-8 PMC10700130

[11] Ouyang, Y. H. et al. Singular dielectric nanolaser with atomic-scale field localization. *Nature* **632**, 287–293 (2024). 39020170 10.1038/s41586-024-07674-9

[12] Li, W. C. et al. Bright optical eigenmode of 1 nm^3^ mode volume. *Phys. Rev. Lett.* **126**, 257401 (2021). 34241506 10.1103/PhysRevLett.126.257401

[13] Yang, Y. X. et al. Generating a nanoscale blade-like optical field in a coupled nanofiber pair. *Photonics Res.* **12**, 154 (2024).

[14] Yang, L. et al. Generating a sub-nanometer-confined optical field in a nanoslit waveguiding mode. *Adv. Photonics* **5**, 046003 (2023).

[15] Wu, H. et al. Photonic nanolaser with extreme optical field confinement. *Phys. Rev. Lett.* **129**, 013902 (2022). 35841559 10.1103/PhysRevLett.129.013902

[16] Wu, H. et al. Plasmonic nanolasers: pursuing extreme lasing conditions on nanoscale. *Adv. Opt. Mater.* **7**, 1900334 (2019).

[17] Wang, F. & Shen, Y. R. General properties of local plasmons in metal nanostructures. *Phys. Rev. Lett.* **97**, 206806 (2006). 17155706 10.1103/PhysRevLett.97.206806

[18] Kuznetsov, A. I. et al. Optically resonant dielectric nanostructures. *Science* **354**, aag2472 (2016). 27856851 10.1126/science.aag2472

[19] Hecht, B. et al. Scanning near-field optical microscopy with aperture probes: fundamentals and applications. *J. Chem. Phys.* **112**, 7761–7774 (2000).

[20] Rotenberg, N. & Kuipers, L. Mapping nanoscale light fields. *Nat. Photonics* **8**, 919–926 (2014).

[21] Eisele, M. et al. Ultrafast multi-terahertz nano-spectroscopy with sub-cycle temporal resolution. *Nat. Photonics* **8**, 841–845 (2014).

[22] Cinchetti, M. et al. Photoemission electron microscopy as a tool for the investigation of optical near fields. *Phys. Rev. Lett.* **95**, 047601 (2005). 16090841 10.1103/PhysRevLett.95.047601

[23] Fitzgerald, J. P. S. et al. Photonic near-field imaging in multiphoton photoemission electron microscopy. *Phys. Rev. B* **87**, 205419 (2013).

[24] Li, Y. L. et al. Revealing low-loss dielectric near-field modes of hexagonal boron nitride by photoemission electron microscopy. *Nat. Commun.* **14**, 4837 (2023). 37563183 10.1038/s41467-023-40603-4 PMC10415285

[25] Fitzgerald, J. P. S., Word, R. C. & Könenkamp, R. Subwavelength visualization of light in thin film waveguides with photoelectrons. *Phys. Rev. B* **89**, 195129 (2014).

[26] Yang, L. et al. Weak-disturbance imaging and characterization of ultra-confined optical near fields. *Light Sci. Appl.* **14**, 358 (2025). 41044060 10.1038/s41377-025-01951-6 PMC12494941

[27] Cotte, Y. et al. Marker-free phase nanoscopy. *Nat. Photonics* **7**, 113–117 (2013).

[28] Kim, T. et al. White-light diffraction tomography of unlabelled live cells. *Nat. Photonics* **8**, 256–263 (2014).

[29] Weiß, S. et al. Exploring three-dimensional orbital imaging with energy-dependent photoemission tomography. *Nat. Commun.* **6**, 8287 (2015). 26437297 10.1038/ncomms9287 PMC4600719

[30] Fukumoto, K. et al. Direct imaging of electron recombination and transport on a semiconductor surface by femtosecond time-resolved photoemission electron microscopy. *Appl. Phys. Lett.* **104**, 053117 (2014).

[31] Fukumoto, K. et al. Femtosecond time-resolved photoemission electron microscopy for spatiotemporal imaging of photogenerated carrier dynamics in semiconductors. *Rev. Sci. Instrum.* **85**, 083705 (2014). 25173274 10.1063/1.4893484

[32] Oh, C. et al. Extending rytov approximation to vector waves for tomography of anisotropic materials. *Phys. Rev. Lett.* **134**, 068101 (2025). 40021185 10.1103/PhysRevLett.134.068101

[33] Bennecke, W. et al. Table-top three-dimensional photoemission orbital tomography with a femtosecond extreme ultraviolet light source. Print at https://arxiv.org/abs/2502.18269 (2025). 10.1038/s41467-026-74308-1 PMC13282482 42321168

[34] Dinh, T. L. et al. A minimalist approach to 3D photoemission orbital tomography: algorithms and data requirements. *New J. Phys.* **26**, 043024 (2024).

[35] Rivenson, Y. et al. Phase recovery and holographic image reconstruction using deep learning in neural networks. *Light Sci. Appl.* **7**, 17141 (2018). 30839514 10.1038/lsa.2017.141 PMC6060068

[36] Rivenson, Y. et al. Deep learning microscopy. *Optica* **4**, 1437–1443 (2017).

